# Curcumin Alleviates Ochratoxin A-Induced Oxidative Injury in Porcine Kidney Epithelial Cells in Association with Restored Nrf2/NQO1/HO-1-Related Antioxidant Expression

**DOI:** 10.3390/antiox15101362

**Published:** 2026-10-21

**Authors:** Hongjie Cui, Yingyi Wu, Chuhan Shao, Cheng Zhang, Shuying Lin, Kaizhao Zhang, Xiaohong Huang

**Affiliations:** 1 Fujian Key Laboratory of Traditional Chinese Veterinary Medicine and Animal Health, College of Animal Sciences, Fujian Agriculture and Forestry University, Fuzhou 350002, China; 2 University Key Laboratory for Integrated Chinese Traditional and Western Veterinary Medicine and Animal Healthcare in Fujian Province, Fujian Agriculture and Forestry University, Fuzhou 350002, China

**Keywords:** mycotoxin nephrotoxicity, PK-15 cells, cytoprotection, redox homeostasis, mitochondrial membrane potential, dietary polyphenols

## Abstract

Ochratoxin A (OTA) damages renal epithelial cells and disrupts redox homeostasis. We examined whether curcumin (Cur) reduces OTA-induced injury in porcine kidney epithelial PK-15 cells and whether protection accompanies changes in antioxidant expression. Preliminary concentration screening gave an OTA half-maximal inhibitory concentration (IC50) of 10.83 μg/mL. Cells received OTA (8 μg/mL), Cur (10 μmol/L), their combination, or control treatment. Cell viability, apoptosis, oxidant burden, mitochondrial membrane potential, and biochemical markers were assessed alongside quantitative reverse-transcription PCR (qRT-PCR) and Western blotting. A previously published RNA-sequencing dataset was reanalyzed to examine redox-related responses. OTA reduced viability and mitochondrial membrane potential, increased apoptosis, and impaired antioxidant defenses. Cur attenuated these changes, lowered oxidant-associated fluorescence, malondialdehyde, and lactate dehydrogenase release, and improved glutathione status. The shared RNA-sequencing dataset contained 11,707 differentially expressed genes in OTA versus control and 498 in OTA+Cur versus OTA, with 380 genes common to both comparisons. Targeted assays showed higher Nrf2, NQO1, and HO-1 mRNA and total-protein abundance with Cur than with OTA alone. These results associate Cur-mediated protection with recovery of antioxidant expression in PK-15 cells. They do not establish Nrf2 activation or a causal requirement for this pathway.

## 1. Introduction

Contamination of food and feed with ochratoxin A (OTA), a fungal metabolite produced by Aspergillus and Penicillium species, raises concerns for both animal health and food safety [1,2,3]. The kidney is a major target of OTA toxicity. Renal exposure can impair tubular epithelial function through several processes, including oxidative damage and disrupted protein homeostasis [2,4]. This makes renal epithelial cells useful for studying cellular responses to OTA and assessing compounds that may reduce injury.

Oxidative stress contributes to OTA nephrotoxicity, although its effects vary with dose, exposure duration, and cell type [4,5,6,7]. One relevant defense system is regulated by nuclear factor erythroid 2-related factor 2 (Nrf2). Under basal conditions, Kelch-like ECH-associated protein 1 (Keap1) promotes Nrf2 degradation. Oxidative or electrophilic stress can disrupt this regulation, allowing Nrf2 to accumulate and stimulate antioxidant response element (ARE)-dependent transcription [8,9,10]. Its target genes include NAD(P)H quinone oxidoreductase 1 (NQO1) and heme oxygenase 1 (HO-1). NQO1 participates in quinone metabolism, whereas HO-1 degrades heme. Their contribution to injury depends on the biological context; in particular, HO-1 expression alone is not a specific indicator of protection or ferroptosis [11,12,13].

Curcumin (Cur), a polyphenol found in Curcuma rhizomes, has been studied for its effects on redox regulation and inflammation [14,15,16]. We selected Cur because these properties are relevant to OTA toxicity and because our earlier work examined Cur-associated changes in apoptosis and PI3K/AKT/mTOR-related expression in PK-15 cells [17]. Evidence from other antioxidant interventions also supports examining redox responses in OTA-exposed renal models [18,19,20]. The present study addresses antioxidant-related expression rather than testing whether Cur is more effective than other polyphenols.

**Figure 1:**
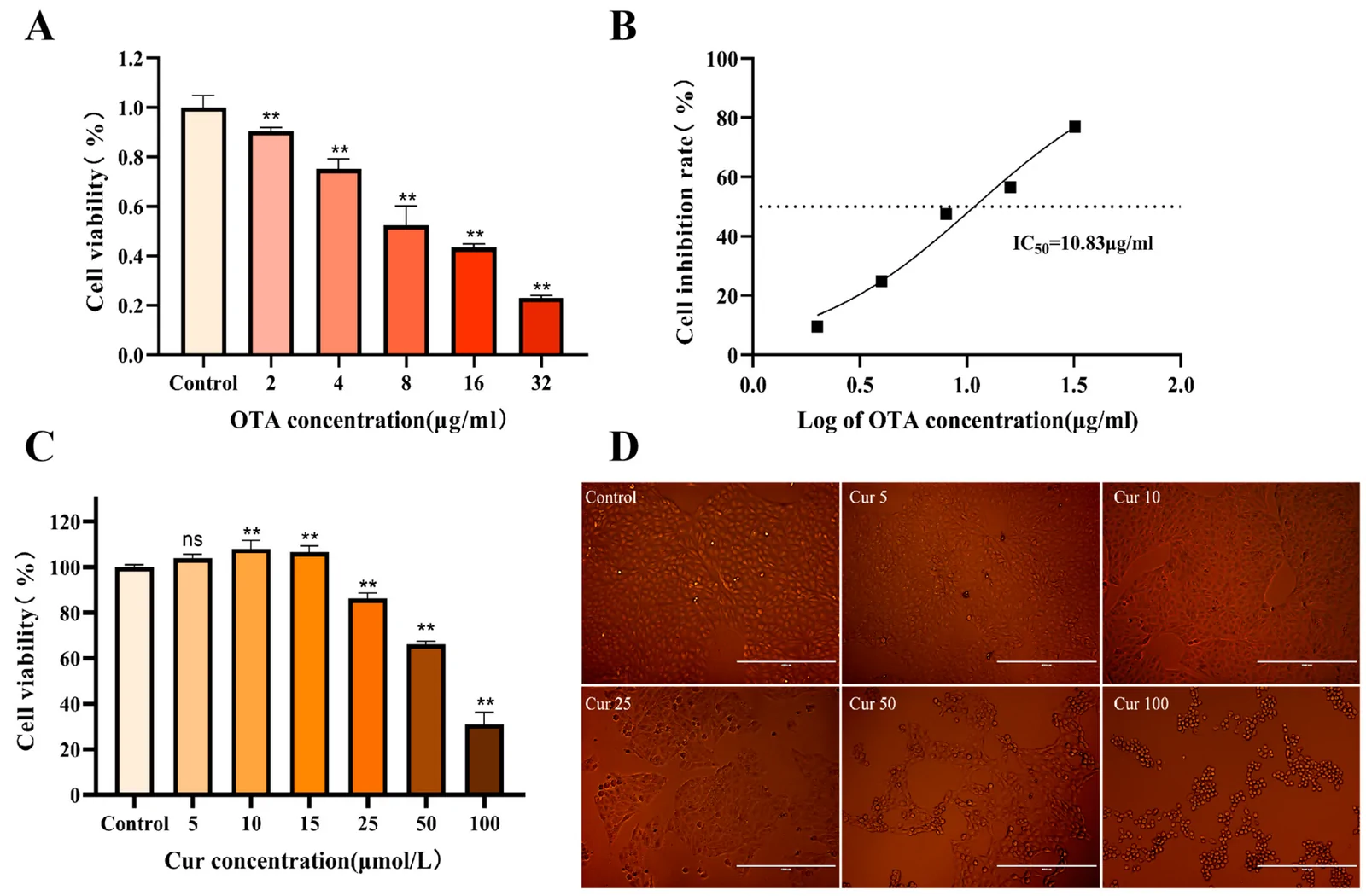
Selection of OTA and Cur treatment concentrations. (**A**) Relative cell viability after treatment with 0–32 μg/mL OTA; (**B**) OTA concentration–inhibition curve and estimated IC50; (**C**) relative cell viability after treatment with 0–100 μmol/L Cur; (**D**) representative cell morphology at different Cur concentrations. Panels (**A**–**C**) are reproduced from Wu et al. [17] and show previously published concentration-screening results; they are not new experiments. Scale bars are shown in the panels. Data are presented as mean ± SD from six replicate wells per group in each experiment; experiments were independently repeated at least three times. ** *p* < 0.01 versus the control group; ns, not significant.

We investigated whether Cur-associated changes in cell injury were accompanied by changes in antioxidant enzyme activity, glutathione status, and Nrf2/NQO1/HO-1 expression. The concentration-screening data in Figure 1A–C and the RNA-sequencing dataset were shared with our previous publication [17]; the latter was reanalyzed here with a focus on redox-related processes. The targeted antioxidant assays address a different question from the earlier PI3K/AKT/mTOR analysis. Whole-cell RNA and protein measurements were used to assess expression, so the study was designed to examine associations with protection rather than to establish Nrf2 activation or pathway necessity.

## 2. Materials and Methods

### 2.1. Cell Culture and Treatment

PK-15 cells (American Type Culture Collection, Manassas, VA, USA) were grown at 37 °C under 5% CO_2_ in high-glucose Dulbecco’s modified Eagle medium (DMEM) containing 10% fetal bovine serum (FBS) and 1% penicillin–streptomycin (all from HyClone, Logan, UT, USA). Treatments began at approximately 70–80% confluence. Stock solutions of OTA (purity ≥ 98%; molecular weight 403.81 g/mol; Acmec Biochemical Co., Ltd., Shanghai, China) and Cur (purity ≥ 94%; Sigma-Aldrich, St. Louis, MO, USA) were prepared in dimethyl sulfoxide (DMSO) and diluted into culture medium before use. The final DMSO concentration was matched across groups and was no greater than 0.1% (*v*/*v*). The four groups were control, OTA (8 μg/mL, approximately 19.8 μmol/L), Cur (10 μmol/L), and OTA+Cur. In the combined-treatment group, cells received Cur for 2 h before OTA was added for a further 24 h. Other treatments lasted 24 h unless stated otherwise. An independent experiment used a separately cultured and treated cell preparation. Technical replicates were repeated measurements within that experiment and did not constitute additional independent experiments. Replicate numbers and technical measurements are specified for each assay below.

### 2.2. CCK-8 Assay for Cell Viability

For the Cell Counting Kit-8 (CCK-8) assay, 96-well plates were seeded with 1 × 10^5^ PK-15 cells per well and incubated for 24 h before treatment. Concentration screening used OTA at 0, 2, 4, 8, 16, or 32 μg/mL and Cur at 0, 5, 10, 15, 25, 50, or 100 μmol/L for 24 h. The previously published screening data are reproduced in Figure 1A–C to explain dose selection [17]. Protection was evaluated after 2 h of Cur pretreatment followed by 24 h of OTA exposure. After a phosphate-buffered saline (PBS) wash, each well received 10 μL CCK-8 reagent (Abbkine, Wuhan, China; KTA1020). Plates were kept at 37 °C in the dark for 2 h before absorbance was read at 450 nm on a SpectraMax Mini reader (Molecular Devices, San Jose, CA, USA). Viability was expressed relative to the control absorbance, and the OTA IC50 was estimated by nonlinear regression in GraphPad Prism version 10.1.2. Six parallel wells were measured per group within each experiment, and the assay was repeated on at least three independent occasions.

### 2.3. Assessment of Cell Morphology

An inverted microscope was used to examine cell attachment, density, shape, and intercellular spacing after treatment. Images were acquired using the same settings across groups. Six wells per group were examined in each experiment, with at least three independent experiments.

### 2.4. Measurement of Intracellular Oxidant Burden

Intracellular oxidant-associated fluorescence was assessed with the CheKine ROS Detection Fluorometric Assay Kit (Green Fluorescence; Abbkine; KTB1910). After treatment, cells were loaded with 2′,7′-dichlorodihydrofluorescein diacetate (DCFH-DA) at 37 °C in the dark using the manufacturer’s recommended probe preparation and incubation procedure. Extracellular probe was removed by washing before microscopy and flow cytometry. ImageJ (version 1.54u8; https://imagej.net/ij/; accessed on 17 October 2026) was used to quantify microscopic fluorescence. The DCFH-DA signal was interpreted as a relative measure of intracellular oxidant burden, not as a measurement of a specific reactive oxygen species (ROS). Six parallel wells or cell samples per group were assessed within each experiment, with at least three independent experiments.

### 2.5. Assessment of Apoptosis and Mitochondrial Membrane Potential

Apoptosis was measured with an Annexin V–fluorescein isothiocyanate (FITC)/propidium iodide (PI) kit (Abbkine; KTA0006). Cells were detached using trypsin without ethylenediaminetetraacetic acid (EDTA), washed in cold PBS, and resuspended in 500 μL of 1× Annexin V binding buffer. Each suspension received 2 μL Annexin V-FITC and 2.5 μL PI, was incubated on ice for approximately 15 min in the dark, and was analyzed by flow cytometry within 30 min after staining, following the kit instructions. A BD FACSCanto II cytometer (BD Biosciences, Franklin Lakes, NJ, USA) recorded at least 10,000 events per sample; FlowJo v10.6 was used for analysis. The apoptotic fraction comprised both Annexin V-positive/PI-negative and Annexin V-positive/PI-positive cells. Mitochondrial membrane potential (ΔΨm) was assessed in separate samples using the JC-1 kit (Abbkine; KTA4001). Cells were incubated with JC-1 working solution for 20 min at 37 °C in the dark, washed twice, and examined by fluorescence microscopy. Green monomer and red aggregate fluorescence were recorded at excitation/emission settings of 488/525 nm and 543/590 nm, respectively. ImageJ was used to calculate the red/green ratio. For JC-1 quantification, one randomly selected field was analyzed from the cell sample obtained in each independent experiment. The independent experiment was the statistical unit (*n* = 3 per group); image fields were not counted as additional independent observations.

### 2.6. Measurement of Oxidative Stress and Cell Injury Markers

Samples were collected after 24 h of treatment. Abbkine kits were used to measure total superoxide dismutase (SOD) activity (KTB1030), catalase (CAT) activity (KTB1040), malondialdehyde (MDA; KTB1052), total and oxidized glutathione (T-GSH and GSSG; KTB1671), and lactate dehydrogenase (LDH) release (KTA1030). Total antioxidant capacity (T-AOC) was assessed by ferric reducing antioxidant power (FRAP) using kit ZC-S0354 (ZCIBIO, Shanghai, China). Assays followed the respective manufacturers’ instructions. Reduced glutathione (GSH) was calculated as T-GSH minus twice the GSSG concentration and used to obtain the GSH/GSSG ratio. For LDH measurement, 100 μL of clarified culture supernatant was mixed with 100 μL of the supplied reaction solution. The reaction was incubated at 37 °C for up to 30 min, and absorbance was recorded at 565 nm with background subtraction, as specified in the kit instructions. Measurements were normalized to protein concentration where required. The SOD assay measured total activity; no selective inhibition step was used to isolate Mn-SOD activity. Six parallel wells or samples per group were measured within each experiment, with at least three independent experiments.

### 2.7. RNA Sequencing and Bioinformatic Analysis

The RNA-sequencing data were generated for our earlier study [17] and reanalyzed here to examine antioxidant and redox-related responses. No new sequencing cohort was generated for this analysis. The dataset comprises three biological samples per group from control, OTA, and OTA+Cur cultures. As described previously, total RNA was isolated using TRIzol (Invitrogen, Carlsbad, CA, USA), assessed for concentration, purity, and integrity, and sequenced by Shanghai Majorbio Bio-pharm Technology Co., Ltd. (Shanghai, China) on an Illumina NovaSeq X Plus platform (Illumina, San Diego, CA, USA). The Majorbio Cloud Platform (https://cloud.majorbio.com/; accessed on 17 October 2026) was used for read quality control, reference-genome alignment, and expression quantification. Differentially expressed genes (DEGs) were defined by an absolute log2 fold change ≥ 1 and a false discovery rate (FDR) < 0.05. Principal component analysis (PCA) summarized sample-level expression patterns. Gene Ontology (GO) and Kyoto Encyclopedia of Genes and Genomes (KEGG) analyses were used to explore functional annotations. Enrichment was interpreted separately for each comparison according to its adjusted *p* value; display in a pathway plot was not taken to imply statistical significance.

### 2.8. qRT-PCR Analysis of Gene Expression

RNA from independently cultured and treated cells was extracted using a commercial kit (Promega, Beijing, China) and converted to complementary DNA (cDNA). Quantitative reverse-transcription PCR (qRT-PCR) was performed on a QuantStudio 5 instrument (Thermo Fisher Scientific, Waltham, MA, USA) with TB Green Premix Ex Taq II (TaKaRa, Dalian, China). NFE2L2 (Nrf2), NQO1, HMOX1 (HO-1), CAT, and SOD2 (Mn-SOD) expression was normalized to ACTB (β-actin) and calculated by the 2^−ΔΔCt^ method. Table 1 lists the primers, porcine transcript accessions, and expected amplicon lengths. Each RNA preparation was measured in six technical replicates. Technical measurements were averaged within each sample before statistical analysis. The qRT-PCR assay included three independent experiments per group, each involving separate cell culture, treatment, and RNA extraction. The independent experiment was the statistical unit (*n* = 3 per group).

### 2.9. Western Blot Analysis

Whole-cell lysates were prepared in radioimmunoprecipitation assay (RIPA) buffer supplemented with protease/phosphatase inhibitors (Roche, Basel, Switzerland). Protein was quantified with a bicinchoninic acid (BCA) kit (Thermo Fisher Scientific, Waltham, MA, USA). Samples containing 30 μg protein were resolved by sodium dodecyl sulfate–polyacrylamide gel electrophoresis (SDS-PAGE) and transferred to polyvinylidene difluoride (PVDF) membranes. After blocking in 5% non-fat milk, membranes were incubated overnight at 4 °C with rabbit primary antibodies against Nrf2 (Immunoway, Suzhou, China; YM8624; clone PT0855R), β-actin (Immunoway, Suzhou, China; YM8343; clone PT0519R), NQO1 (UpingBio, Hangzhou, China; YP-Ab-02723), or HO-1 (UpingBio, Hangzhou, China; YP-Ab-18677), each at 1:1000. A horseradish peroxidase (HRP)-conjugated goat anti-rabbit IgG (H+L) secondary antibody (Immunoway, Suzhou, China; RS0002) was used at 1:5000. Enhanced chemiluminescence (ECL) signals were quantified in ImageJ after background subtraction, and each target was normalized to β-actin. Densitometry used one protein lysate per group from each of three independent experiments involving separately cultured and treated cells. The independent experiment was the statistical unit (*n* = 3 per group). These measurements represent whole-cell protein abundance and do not resolve nuclear and cytoplasmic Nrf2.

### 2.10. Statistical Analysis

Results are reported as means with standard deviations (SDs). Group comparisons used one-way analysis of variance (ANOVA) with Tukey’s multiple-comparisons test; two-tailed Student’s *t*-tests were used for two-group comparisons where applicable. Distributional assumptions and variance homogeneity were assessed before parametric testing. Statistical significance was set at *p* < 0.05. GraphPad Prism version 10.1.2 was used for analysis and graph preparation. For qRT-PCR, Western blotting, and JC-1 quantification, the independent experiment was the statistical unit (*n* = 3 per group). Technical qRT-PCR replicates were averaged within each sample before group statistics were calculated. Technical reactions and image fields did not increase *n*. For RNA-seq, each independently prepared RNA sample was the statistical unit (*n* = 3 per group). Asterisks in the figures denote comparisons with control, and number signs denote comparisons with OTA.

## 3. Results

### 3.1. Curcumin Attenuates OTA-Induced Reductions in PK-15 Cell Viability, Morphological Injury, and Apoptosis

The previously published concentration screen [17] was used to select a submaximal OTA exposure for the antioxidant experiments. OTA reduced viability in a concentration-dependent manner over 0–32 μg/mL. Inhibition rates were 9.57% and 76.99% at 2 and 32 μg/mL OTA, respectively (Figure 1A), and the fitted IC50 was 10.83 μg/mL (Figure 1B). OTA at 8 μg/mL was selected because it produced measurable injury below the IC50 while retaining sufficient viable cells for analyses of oxidative stress, mitochondrial function, and molecular expression.

Cur at 5–15 μmol/L did not reduce viability below control levels, although the plotted values at 10 and 15 μmol/L were significantly above control. Viability decreased significantly at 25 μmol/L and declined further at 50 and 100 μmol/L (Figure 1C). Consistent with these findings, reduced cell density and altered morphology were evident at 25 μmol/L, whereas rounding, increased intercellular gaps, and detachment were more pronounced at 50 and 100 μmol/L (Figure 1D). Based on cell tolerance and the subsequent protective-dose screening, 10 μmol/L Cur was selected because it was well tolerated and provided a relatively strong protective effect.

**Figure 2:**
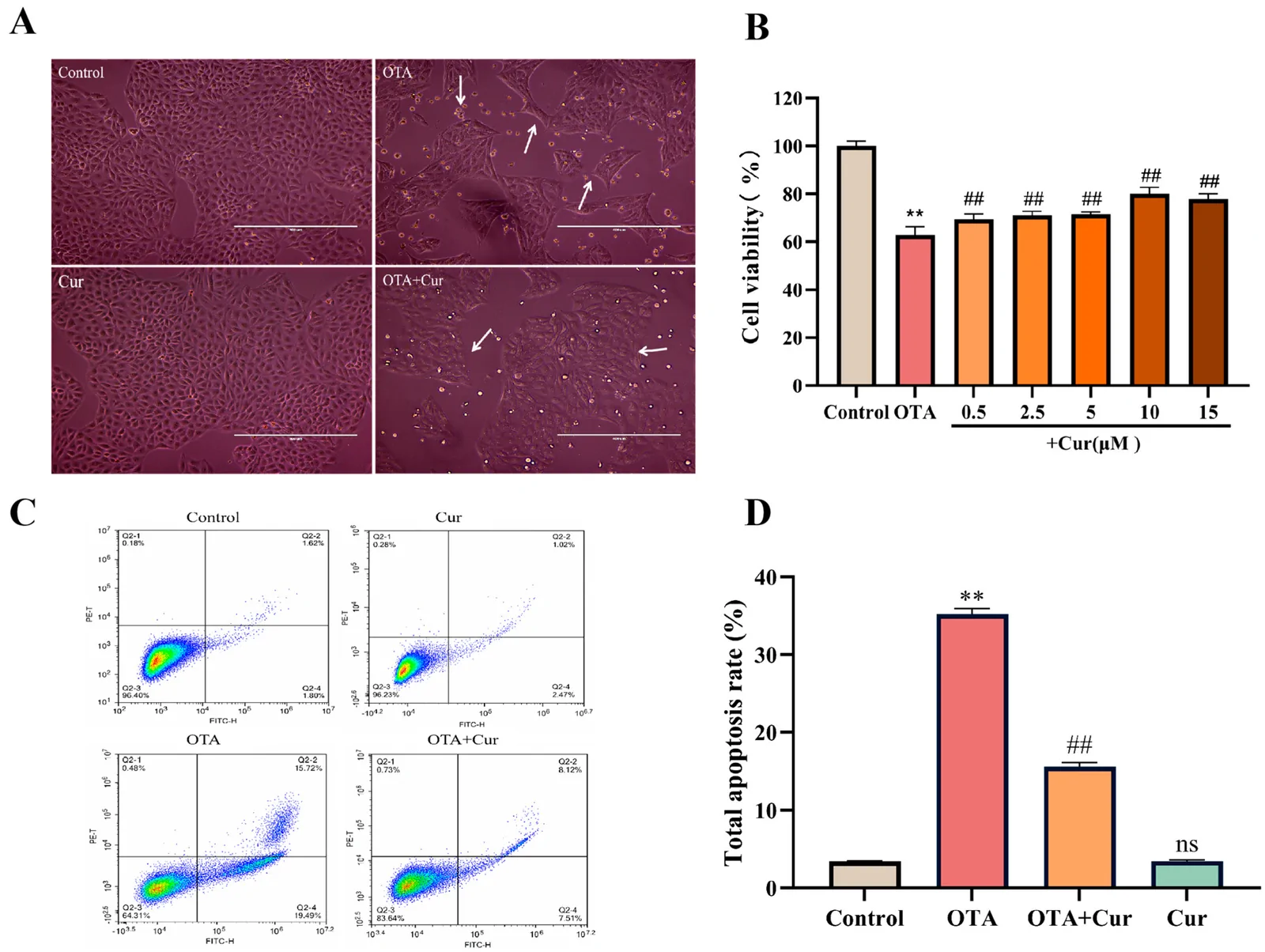
Effects of Cur on OTA-induced cytotoxicity and apoptosis. (**A**) Representative morphological images of the control, OTA, Cur, and OTA+Cur groups. White arrows indicate cells showing morphological alterations, including rounding and shrinkage; (**B**) cell viability after OTA exposure with 0.5, 2.5, 5, 10, or 15 μmol/L Cur; (**C**) flow cytometric analysis of apoptosis; (**D**) total apoptotic rate. Data are presented as mean ± SD from six replicate samples per group in each experiment; experiments were independently repeated at least three times. ** *p* < 0.01 versus the control group; ## *p* < 0.01 versus the OTA group; ns, not significant.

In the four-group experiment, control cells were densely adherent with well-defined borders. OTA markedly reduced cell density and induced rounding, shrinkage, and detachment, consistent with cytotoxicity. Cells treated with Cur alone resembled controls, indicating that 10 μmol/L Cur did not adversely affect cell morphology. The OTA+Cur group showed improved adhesion and spreading, with fewer rounded and detached cells than the OTA group (Figure 2A). Protective-dose screening showed that 0.5–15 μmol/L Cur increased viability to varying degrees following OTA exposure, with the greatest recovery at 10 and 15 μmol/L (Figure 2B). Flow cytometry showed that OTA significantly increased early, late, and total apoptosis, whereas Cur markedly reduced OTA-induced apoptosis (Figure 2C,D).

### 3.2. Curcumin Reduces OTA-Induced ROS Accumulation and Loss of Mitochondrial Membrane Potential

**Figure 3:**
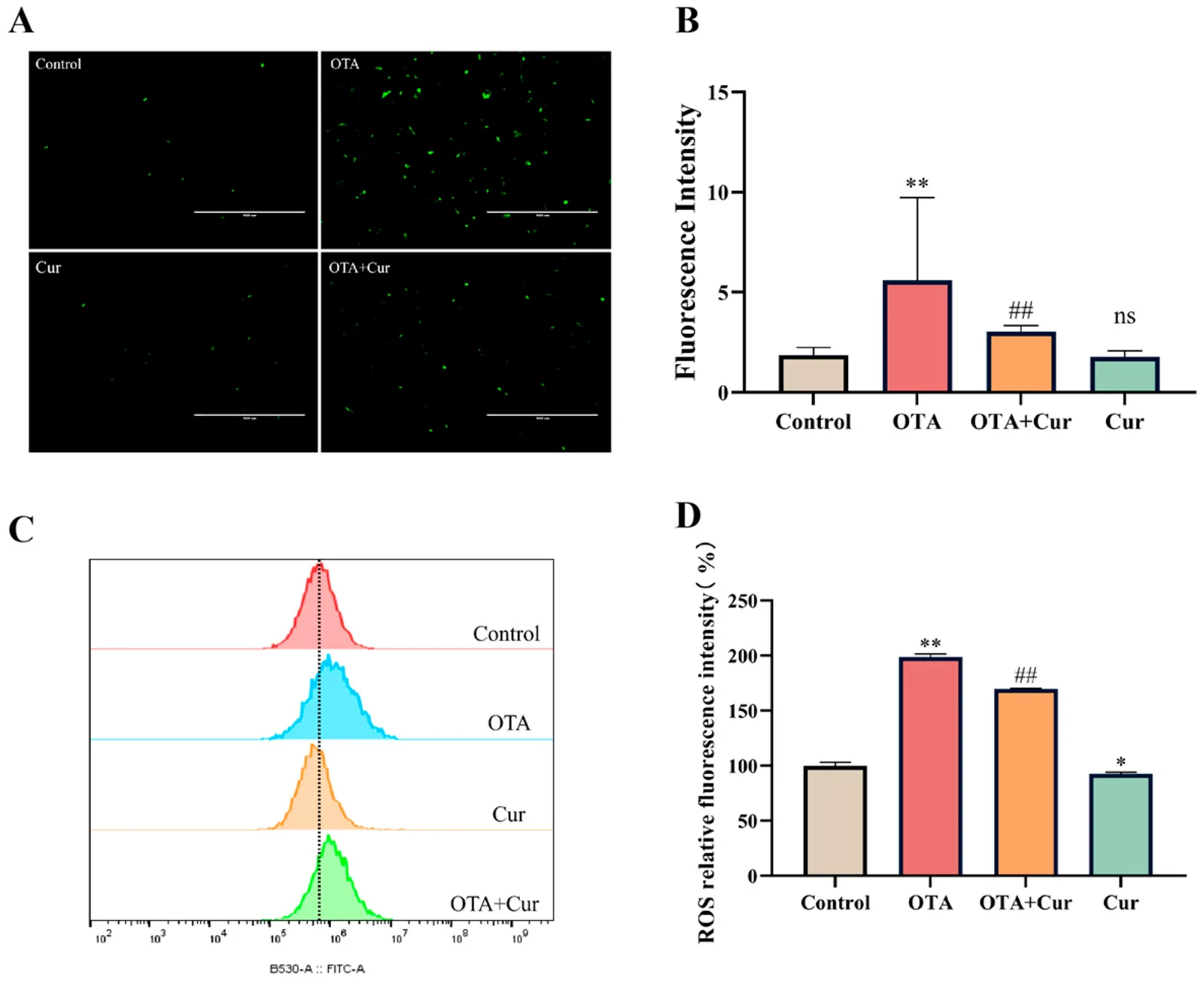
Cur reduces OTA-induced intracellular ROS accumulation. (**A**) DCFH-DA fluorescence images; (**B**) fluorescence intensity in microscopic images; (**C**) fluorescence distribution measured by flow cytometry; (**D**) relative ROS fluorescence intensity measured by flow cytometry. The group order is Control, OTA, Cur, and OTA+Cur in (**A**,**C**), and Control, OTA, OTA+Cur, and Cur in (**B**) and (**D**). Scale bars are shown in (**A**). Data are presented as mean ± SD from six replicate samples per group in each experiment; experiments were independently repeated at least three times. * *p* < 0.05 and ** *p* < 0.01 versus the control group; ## *p* < 0.01 versus the OTA group; ns, not significant.

OTA-treated cells showed stronger DCFH-DA fluorescence than control or Cur-treated cells. Fluorescence was lower with OTA+Cur than with OTA alone (Figure 3A,B), indicating a reduction in the oxidant-associated signal.

Flow cytometry gave the same direction of change: OTA increased fluorescence relative to control, and Cur reduced it in OTA-exposed cells (Figure 3C,D). These measurements support a change in intracellular oxidant burden but do not identify the ROS or cellular source.

**Figure 4:**
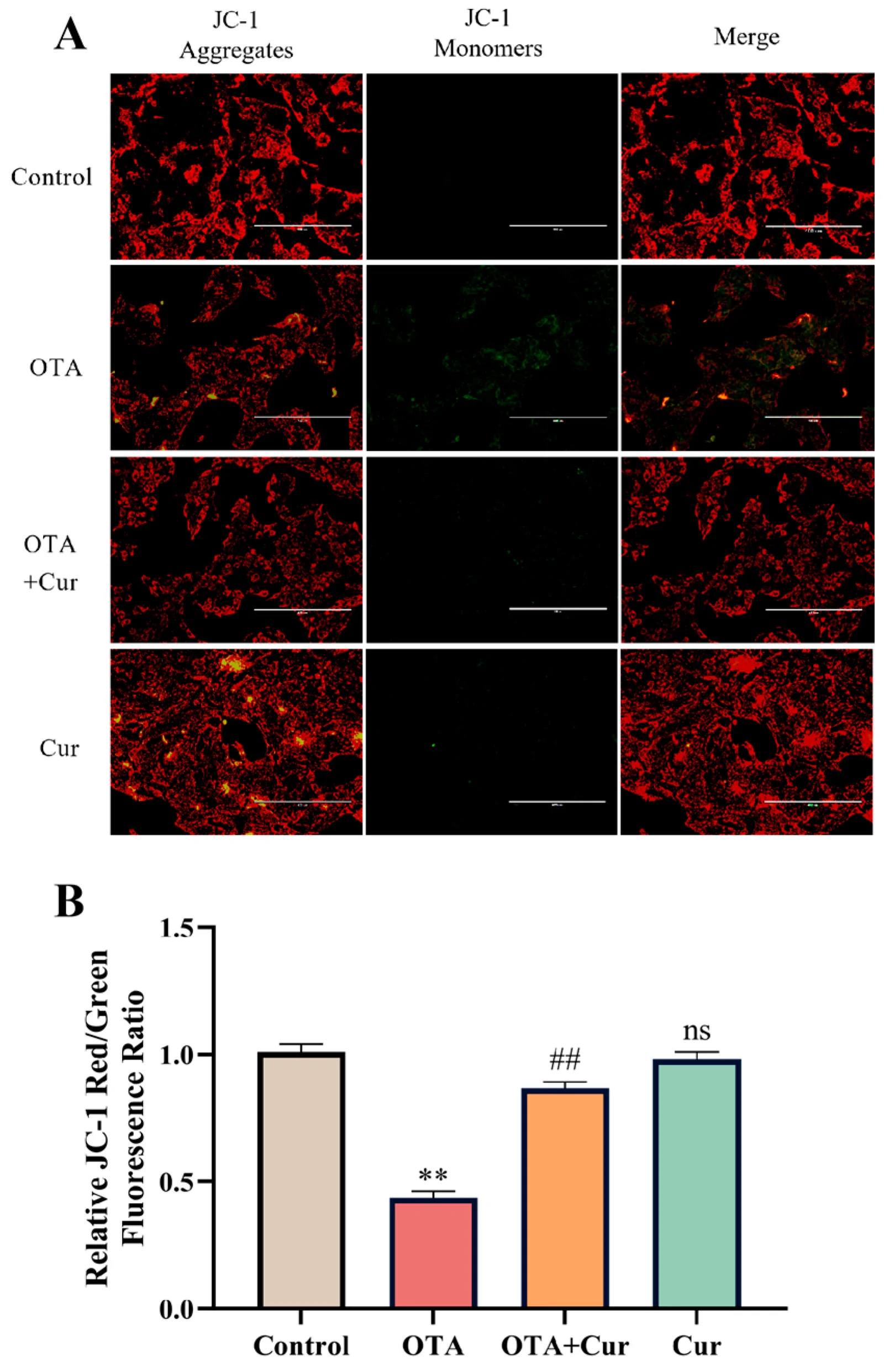
Cur attenuates OTA-induced loss of mitochondrial membrane potential. (**A**) JC-1 fluorescence images showing aggregates (red), monomers (green), and merged signals; (**B**) JC-1 red/green fluorescence ratio. Scale bars are shown in the panels. Data are mean ± SD from three independent experiments per group (*n* = 3). One randomly selected field was analyzed from each independently cultured sample; the independent experiment was the statistical unit. ** *p* < 0.01 versus the control group; ## *p* < 0.01 versus the OTA group; ns, not significant.

JC-1 staining showed that OTA decreased red aggregate fluorescence, increased green monomer fluorescence, and significantly reduced the red/green fluorescence ratio, indicating mitochondrial depolarization. Relative to OTA alone, Cur increased red fluorescence, decreased green fluorescence, and significantly increased the red/green ratio (Figure 4A,B). Thus, ROS accumulation coincided with loss of mitochondrial membrane potential after OTA exposure, whereas Cur treatment was associated with lower ROS and improved membrane potential. These observations do not establish the direction of causality between ROS accumulation and mitochondrial changes.

### 3.3. Curcumin Attenuates OTA-Induced Impairment of Antioxidant Capacity and Cellular Injury

**Figure 5:**
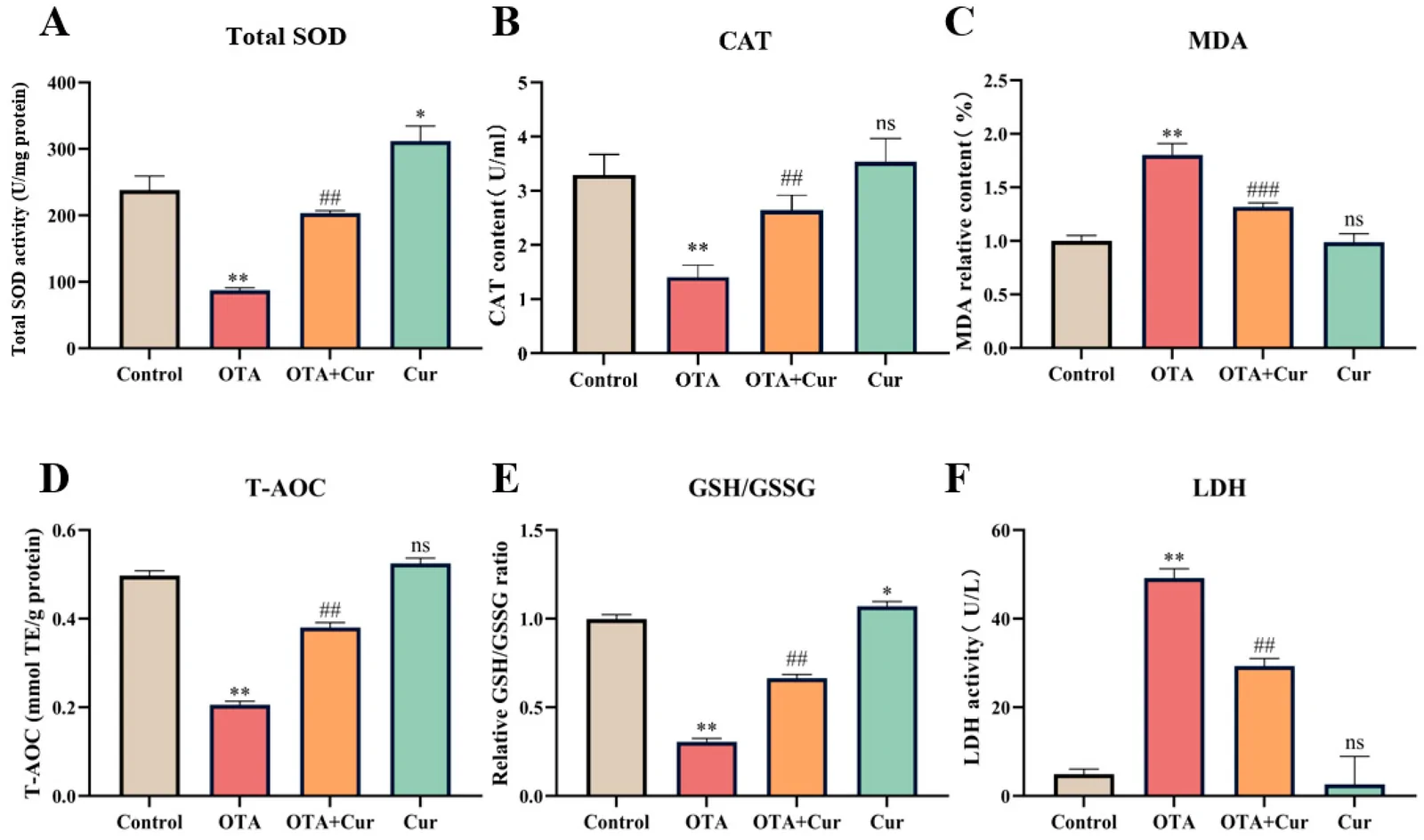
Cur attenuates OTA-induced redox imbalance and cell injury. (**A**) total SOD activity; (**B**) CAT activity; (**C**) relative MDA content; (**D**) T-AOC; (**E**) GSH/GSSG ratio; (**F**) LDH activity. Data are presented as mean ± SD from six replicate samples per group in each experiment; experiments were independently repeated at least three times. * *p* < 0.05 and ** *p* < 0.01 versus the control group; ## *p* < 0.01 and ### *p* < 0.001 versus the OTA group; ns, not significant.

OTA lowered total SOD and CAT activities, T-AOC, and the GSH/GSSG ratio relative to control. MDA and extracellular LDH were higher after OTA exposure (Figure 5A–F). These changes indicate impaired antioxidant capacity together with greater lipid peroxidation and membrane injury.

Cur increased total SOD and CAT activities, T-AOC, and the GSH/GSSG ratio in OTA-exposed cells, while reducing MDA and LDH (Figure 5A–F). The biochemical changes accompanied improvements in viability, apoptosis, and mitochondrial membrane potential.

### 3.4. Transcriptomic Analysis Identifies OTA-Associated Oxidative Stress Responses and Expression Changes with Cur Treatment

**Figure 6:**
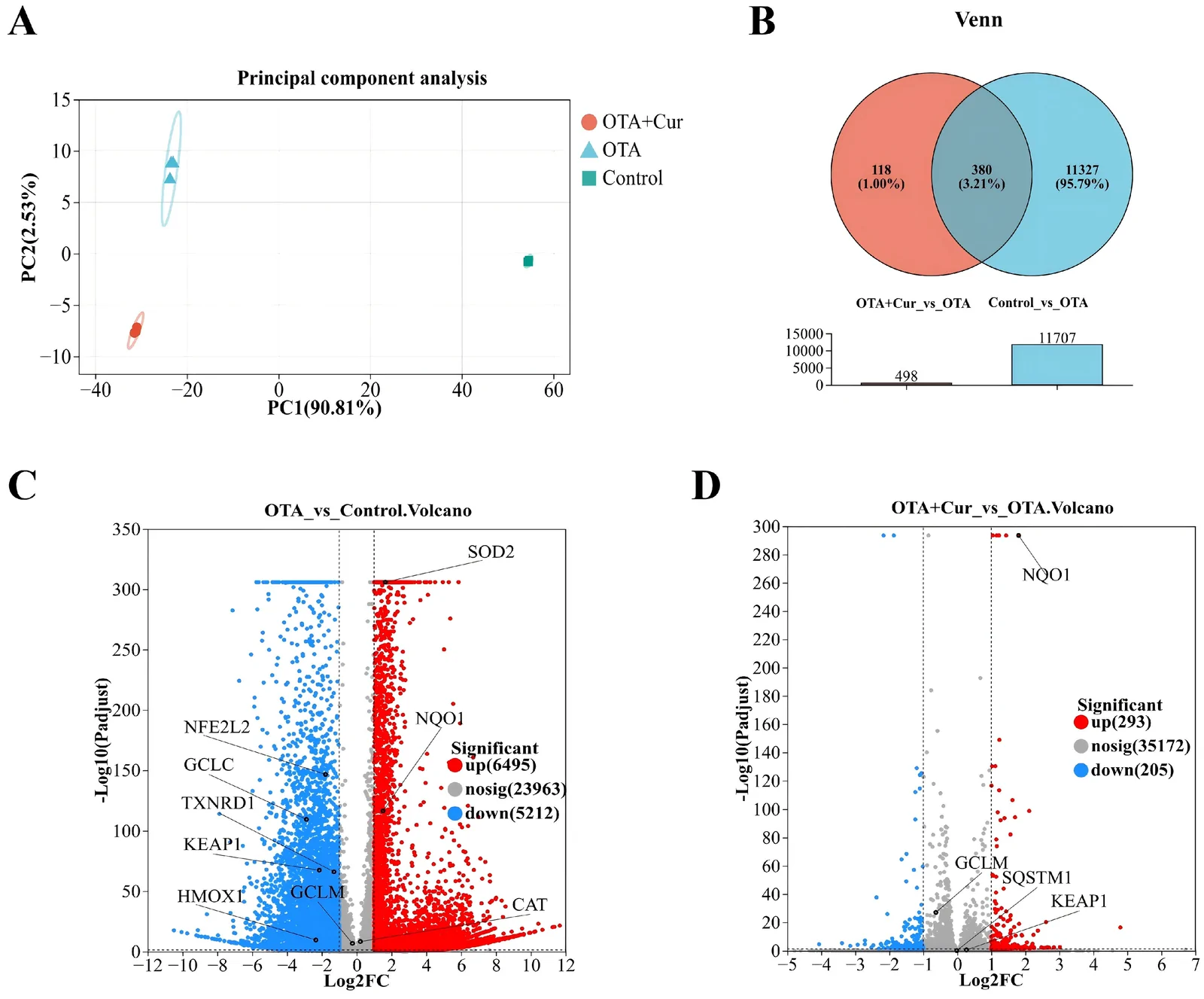

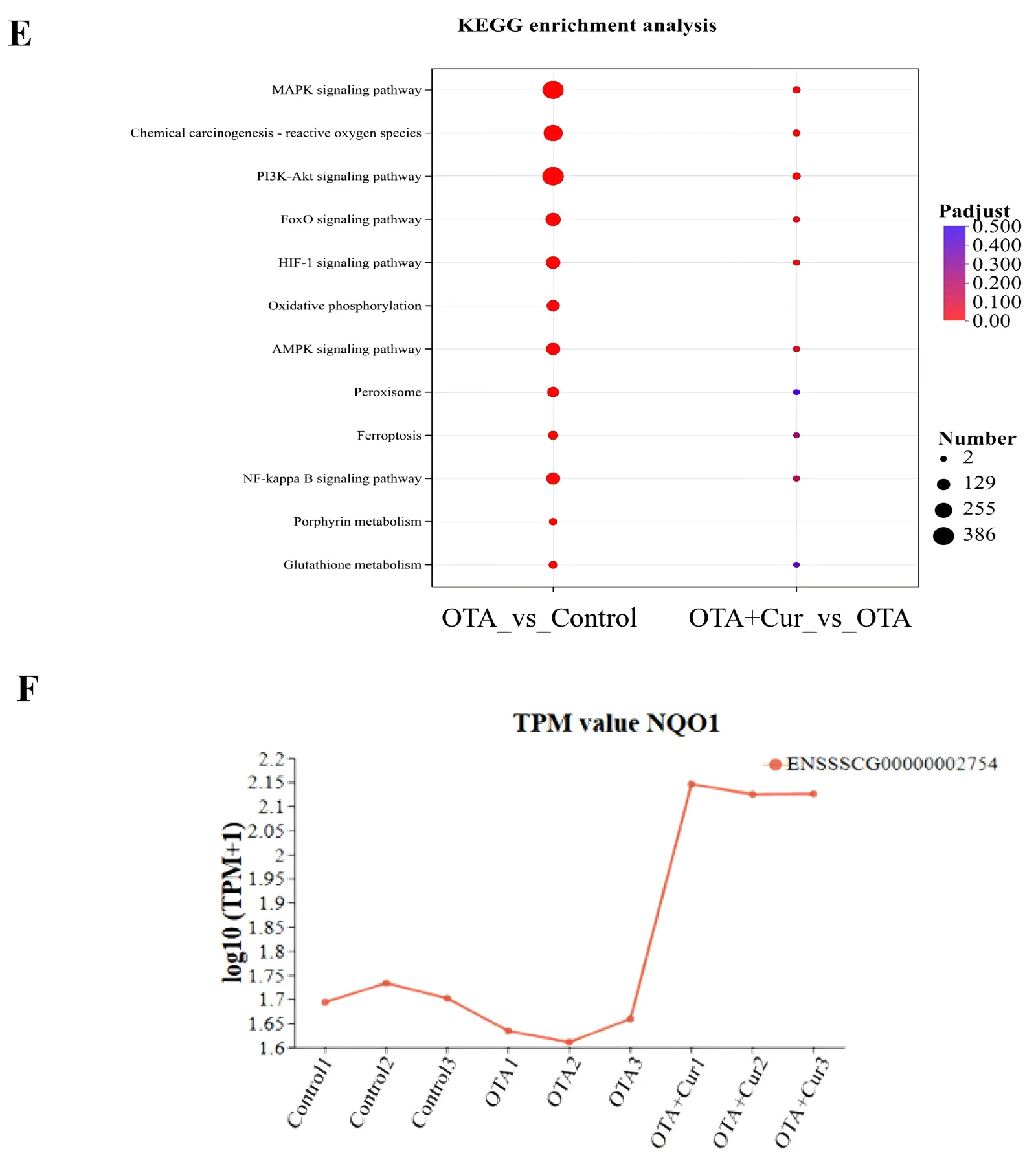
Redox-focused analysis of the RNA-sequencing dataset previously reported by Wu et al. [17]. (**A**) PCA. (**B**) DEG overlap: 11,707 genes in the OTA/control comparison, 498 in OTA+Cur versus OTA, and 380 in both sets. The Control_vs_OTA label in the Venn diagram denotes the same pair of groups as C; reversing a comparison does not change DEG-set membership. (**C**) OTA versus control: 6495 upregulated and 5212 downregulated genes. (**D**) OTA+Cur versus OTA: 293 upregulated and 205 downregulated genes. Positive log2 fold changes indicate higher expression in the first-named group. (**E**) KEGG annotations for each comparison; bubble size indicates gene count and color indicates adjusted *p* value. Not all displayed pathways meet the significance threshold. (**F**) NQO1 expression as log10(TPM + 1) in the nine samples. The dataset includes three biological samples per group. Panel (**F**) shows TPM-based relative abundance, whereas (**C**) reports count-based differential expression; the distinction is described in Section 3.4.

In the shared RNA-sequencing dataset [17], PCA separated the control, OTA, and OTA+Cur groups along the first two principal components, which explained 90.81% and 2.53% of the variance, respectively (Figure 6A). PC1 accounted for most of the observed variation, with OTA exposure associated with the largest transcriptomic difference; Cur-treated samples also exhibited a profile distinct from OTA alone. Differential expression analysis identified 11,707 DEGs in OTA versus control, comprising 6495 upregulated and 5212 downregulated genes. In OTA+Cur versus OTA, 498 DEGs were identified, comprising 293 upregulated and 205 downregulated genes (Figure 6C,D). The two comparisons shared 380 DEGs (Figure 6B). This overlap identifies genes responding in both contrasts but does not establish reversal of the OTA response, which depends on the direction and magnitude of individual gene changes.

The KEGG plots included pathways related to chemical carcinogenesis–reactive oxygen species, glutathione metabolism, oxidative phosphorylation, ferroptosis, and mitogen-activated protein kinase (MAPK) signaling (Figure 6E). Their adjusted *p* values differed between comparisons, and several pathways displayed for OTA+Cur versus OTA did not meet an adjusted *p* < 0.05 threshold. The annotations were therefore treated as exploratory. They do not establish ferroptosis or MAPK-dependent cell death [11,13,21]. NQO1 was higher in OTA+Cur than in OTA in both the differential-expression plot and the displayed transcripts per million (TPM) profile (Figure 6D,F). Although the TPM profile showed lower NQO1 abundance in the OTA group than in controls, count-based differential-expression analysis identified an increase (log2FC = 1.516, adjusted *p* < 0.001). This difference may reflect the distinct normalization and scaling procedures used for abundance visualization and differential-expression analysis.

### 3.5. Curcumin Restores Nrf2/NQO1/HO-1-Related Expression and Antioxidant Defenses

**Figure 7:**
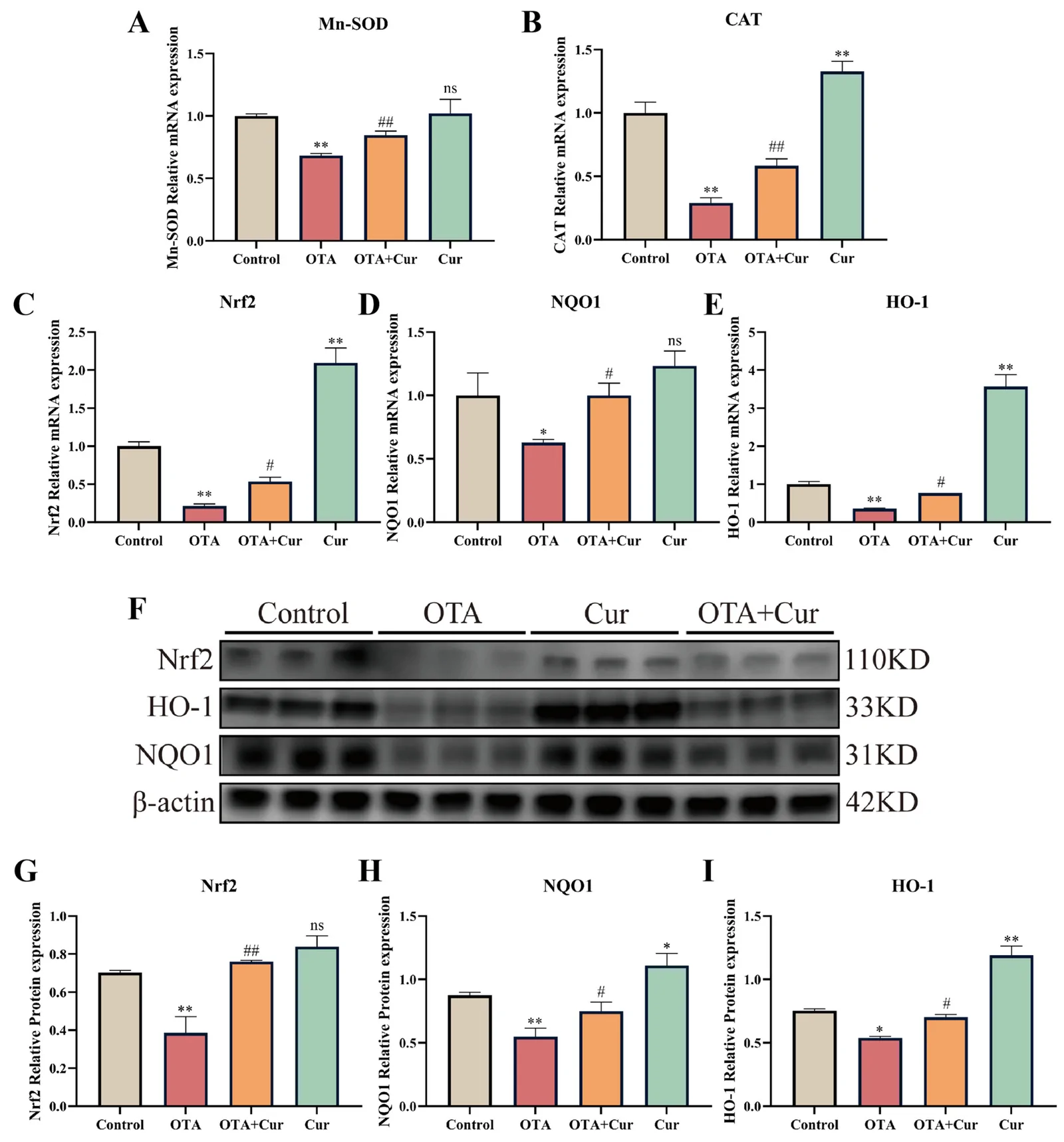
Analysis of antioxidant-related gene and protein expression by qRT-PCR and Western blotting. (**A**–**E**) Relative mRNA expression of Mn-SOD, CAT, Nrf2, NQO1, and HO-1; (**F**) representative bands for Nrf2, HO-1, NQO1, and β-actin, with lanes ordered as Control, OTA, Cur, and OTA+Cur and three lanes per group; (**G**–**I**) densitometric quantification of Nrf2, NQO1, and HO-1. Data are mean ± SD from three independent experiments per group for both qRT-PCR and Western blotting (*n* = 3). Each experiment used separately cultured and treated cells. The six technical qRT-PCR replicates per sample were averaged before calculating group statistics; the independent experiment was the statistical unit. The order of the Western blot lanes in (**F**) differs from the bar order in (**A**–**E**) and (**G**–**I**) (Control, OTA, OTA+Cur, Cur). * *p* < 0.05 and ** *p* < 0.01 versus the control group; # *p* < 0.05 and ## *p* < 0.01 versus the OTA group; ns, not significant.

OTA reduced SOD2 (Mn-SOD), CAT, NFE2L2 (Nrf2), NQO1, and HMOX1 (HO-1) mRNA levels relative to control, whereas Cur increased all five transcripts in OTA-exposed cells (Figure 7A–E). The changes were significant as indicated in the figure. SOD2 expression concerns the mitochondrial SOD isoform; the biochemical assay in Figure 5A measured total SOD activity and cannot attribute that activity specifically to Mn-SOD. The Cur-associated increase in NQO1 was also observed in the RNA-sequencing analysis.

Total Nrf2, NQO1, and HO-1 protein abundance was lower after OTA exposure and higher in the OTA+Cur group than in the OTA group (Figure 7F–I). Thus, recovery of these proteins accompanied lower oxidant burden and improved biochemical and cellular endpoints. The measurements do not determine whether Nrf2 entered the nucleus or whether Nrf2-dependent transcription was required for protection.

## 4. Discussion

Cur reduced OTA-associated injury in PK-15 cells under the conditions tested. Improvements in viability and apoptosis accompanied lower oxidant-associated fluorescence, less mitochondrial depolarization, and recovery of antioxidant measures. Nrf2, NQO1, and HO-1 mRNA and total-protein abundance also increased relative to OTA alone. These observations identify expression changes that accompany protection, but the endpoint design cannot establish their temporal order or causal contribution.

The OTA response is consistent with earlier reports of oxidative damage in renal cells [4,5,7]. DCFH-DA fluorescence increased while the JC-1 red/green ratio declined. Lower total SOD and CAT activities and a lower GSH/GSSG ratio indicate reduced antioxidant capacity, whereas higher MDA and LDH support lipid peroxidation and membrane injury. Mitochondrial dysfunction could contribute to oxidant production, but DCFH-DA is not mitochondria-specific and JC-1 does not measure respiration. The present data therefore do not establish mitochondrial electron leakage as the source of the increased oxidant signal.

The recovery of several injury endpoints with Cur agrees with studies of antioxidant interventions in OTA-exposed renal models [11,18,20]. Recent work in Caco-2 cells also links antioxidant treatment with changes in glutathione-related defenses and Nrf2 regulation during mycotoxin exposure [22], although that intestinal model differs from PK-15 cells. Our earlier study examined apoptosis and PI3K/AKT/mTOR-related expression under similar exposure conditions [17]. The current work extends the redox assessment, but the shared concentration-screening and sequencing data should not be regarded as independent replication of that earlier study.

The transcriptomic analysis must be interpreted in that context. The DEG counts and 380-gene overlap derive from the shared dataset, not from a new sequencing experiment. Overlap between contrasts does not itself demonstrate reversal of injury. The current pathway analysis directs attention to redox and stress responses, but statistical support varies by pathway and comparison. In particular, lipid peroxidation and a ferroptosis annotation are insufficient to diagnose ferroptotic death; iron dependence and functional rescue would need to be tested [11,13,21]. For NQO1, the TPM profile and qRT-PCR showed a lower value after OTA exposure, whereas the count-based model identified upregulation. Differences in normalization may contribute to this divergence, but this explanation has not been tested directly. The evidence for an increase with Cur relative to OTA is consistent across these analyses.

Nrf2-dependent transcription is a plausible contributor to antioxidant adaptation [6,8,9,10]. Nevertheless, increased total Nrf2 protein is not equivalent to activation, and changes in NQO1 or HO-1 need not arise exclusively from Nrf2. HO-1 has shown protective effects in an OTA-exposed mouse model [12], while its relationship with iron-dependent injury is context-dependent [11,13]. To test whether Nrf2 is necessary for Cur-associated protection, future studies should compare injury endpoints after NFE2L2 knockdown, verify knockdown efficiency, and include non-targeting controls. Rescue with an interference-resistant NFE2L2 construct would help assess specificity. Nuclear localization and an ARE reporter could test transcriptional activation, while pharmacological inhibitors would require controls for off-target toxicity.

Several limitations restrict interpretation. The experiments used one porcine cell line and one selected treatment regimen; they do not reproduce chronic dietary exposure in animals. Nuclear Nrf2 localization, ARE activity, and Nrf2 loss-of-function or rescue were not assessed. Mitochondrial ROS and respiratory-chain activity were also not measured. Total SOD activity cannot resolve individual SOD isoforms. The sequencing analysis reused an existing dataset, and its exploratory findings do not independently validate a mechanism. Larger studies and functional interventions are needed to test the contribution of antioxidant expression to Cur-associated protection.

## 5. Conclusions

Cur attenuated OTA-induced oxidative and cellular injury in PK-15 cells, accompanied by recovery of Nrf2, NQO1, and HO-1 expression. Functional experiments are needed to determine whether these changes are required for protection. The findings support further evaluation of Cur in livestock-relevant exposure models, including feed-additive studies that address bioavailability, safety, and efficacy. The concentrations used in cell culture cannot be converted directly into dietary inclusion levels.

## Figures and Tables

**Table 1:** qRT-PCR primer sequences.

Target Gene	Accession No.	Primer Sequence (5′ → 3′)	Product Length (bp)
Nrf2	XM_021075133.2	F: GAAAGCCCAGTCTTCATTGC R: TTGGAACCGTGCTAGTCTCA	190
NQO1	NM_001159613.1	F: CATGGCGGTCAGAAAAGCAC R: ATGGCATACAGGTCCGACAC	135
HO-1	NM_001004027.1	F: TACCGCTCCCGAATGAACAC R: GTCACGGGAGTGGAGTCTTG	209
CAT	NM_214301.2	F: CGCCTATTTGCCTATCCTGACACTC R: GCACGGAAGGGACAGTTCACAG	83
Mn-SOD	NM_214127.2	F: TTTCTGGACAAATCTGAGCCCTAACG R: CGACGGATACAGCGGTCAACTTC	122
β-actin	NM_001444420.1	F: CAAGGACCTCTACGCCAACAC R: TGGAGGCGCGATGATCTT	130

## Data Availability

The RNA-sequencing data analyzed in this study are publicly available in the NCBI Sequence Read Archive under BioProject accession PRJNA1543739 (https://www.ncbi.nlm.nih.gov/sra/PRJNA1543739; accessed on 17 October 2026). The release date specified by NCBI was 14 October 2026. This dataset was previously used in Wu et al. [17] and was reanalyzed here to investigate antioxidant and redox-related responses. No new sequencing data were generated for the present study. Processed data and other supporting experimental data are available from the corresponding author upon reasonable request.
